# Frequency of Oral Lesions, Olfactory, and Gustatory Disorders and Xerostomia in Patients with COVID-19

**DOI:** 10.3390/dj12060179

**Published:** 2024-06-11

**Authors:** Natália Lopes Castilho, Daniella R. Barbosa Martelli, Renato Assis Machado, Zêus Araujo Cunha, Claudiojanes dos Reis, Priscila Regina Queiroz, Dayane de Sá Silva, Eduardo Araujo Oliveira, Ricardo D. Coletta, Hercílio Martelli-Júnior

**Affiliations:** 1 Health Science/Primary Care Postgraduate Program, State University of Montes Claros (UNIMONTES), Montes Claros 39400-000, Brazil; nlcastilho@hotmail.com (N.L.C.); araujocunhazeus@gmail.com (Z.A.C.); 2 Health Science/Primary Care Postgraduate Program, Department of Oral Medicine and Oral Pathology, Dental School, State University of Montes Claros (UNIMONTES), Montes Claros 39400-000, Brazil; daniella.martelli@unimontes.br (D.R.B.M.); hercilio.junior@unimontes.br (H.M.-J.); 3 Department of Oral Diagnosis and Graduate Program in Oral Biology, School of Dentistry, University of Campinas, Piracicaba 13414-018, Brazil; coletta@unicamp.br; 4 Mário Ribeiro Clinical Hospital, School of Medicine, Funorte University Center, Montes Claros 39401-222, Brazil; claudiojanesreis@funorte.edu.br (C.d.R.); priscila.queiroz@soufunorte.com.br (P.R.Q.); dayane.indyara@hcmarioribeiro.com (D.d.S.S.); 5 Health Sciences Postgraduate Program, Department of Pediatrics, School of Medicine, Federal University of Minas Gerais (UFMG), Belo Horizonte 30310-580, Brazil; eduolive@ufmg.br; 6 Department of Pediatrics, Rady Children’s Hospital, University of California, San Diego, CA 92093, USA

**Keywords:** COVID-19, oral lesions, olfactory disorders, gustatory disorders, xerostomia

## Abstract

COVID-19, a respiratory illness with a global impact on millions, has recently been linked to manifestations affecting various bodily systems, including the oral cavity. Studies highlight oral issues, like ulcers, blisters, and white patches, alongside olfactory and gustatory dysfunction, influencing an individual’s quality of life. In this context, our study aimed to assess the frequency of oral lesions, olfactory and gustatory disorders, and xerostomia resulting from COVID-19. An observational study was conducted with 414 patients to evaluate the frequency of oral symptoms resulting from COVID-19. Patients were diagnosed with mild symptoms and evaluated through clinical examination of the oral cavity and a questionnaire to assess functional alterations. The findings showed that 139 out of 414 patients presented clinical manifestations, with oral lesions being the most prevalent (19.1%), followed by gustatory disorders (18.1%), xerostomia (14.2%), and olfactory dysfunction (14%). The most prevalent oral lesions were ulcerations (*n* = 51), candidiasis (*n* = 8), and erythema or red plaques (*n* = 7). Unfortunately, 50 (12.1%) patients died during this study. Therefore, oral lesions, olfactory and gustatory dysfunctions, and xerostomia are common symptoms associated with COVID-19.

## 1. Introduction

As of May 2024, the global tally of reported cases of coronavirus disease (COVID-19) has surged past 775 million, with over 7.0 million documented deaths worldwide [1]. In Brazil alone, the figures stand at approximately 38.8 million confirmed cases and 712,038 reported deaths [2]. Brazil notably emerged as a focal point of the pandemic, bearing one of the most profound impacts globally [3]. Against this backdrop, the COVID-19 pandemic has posed significant challenges in managing patients with chronic illnesses or comorbidities, exacerbating concerns within Brazil’s healthcare landscape. Throughout the COVID-19 era, various conditions have witnessed heightened prevalence rates in Brazil, including but not limited to systemic lupus erythematosus, herpes zoster, and Sjogren’s syndrome [4,5,6]. This amplifies the complexity of healthcare provision, underscoring the urgent need for comprehensive strategies to address the multifaceted impact of the pandemic on public health.

The rapid identification of the oral cavity as a potential site for infection underscores its critical role in the pathogenesis of COVID-19, with implications extending beyond respiratory symptoms alone [7]. This recognition is substantiated by the ability of severe acute respiratory syndrome coronavirus-2 (SARS-CoV-2) to bind to the angiotensin-converting enzyme 2 (ACE2) receptor, a key component of the renin–angiotensin system widely expressed in the oral cavity, including the tongue [8]. Such binding events facilitate viral entry and subsequent replication, setting the stage for local and systemic inflammatory responses. Consequently, the oral cavity emerges as a gateway for viral transmission and disease progression, necessitating a comprehensive understanding of its involvement in the context of COVID-19. Indeed, emerging evidence from multiple studies corroborates the occurrence of oral lesions and related alterations, such as gustatory disorders, olfactory dysfunction, and xerostomia, in individuals afflicted with COVID-19 [9,10,11,12,13].

Olfactory dysfunction represents a complex array of conditions, which can be categorized into conductive losses resulting from obstructions within the nasal passages or sensorineural causes arising from damage to the olfactory neuroepithelium [14]. Among these, postviral olfactory loss stands out as a prominent etiological factor, with respiratory viruses often implicated in its pathogenesis. This form of olfactory dysfunction occurs when viral infections lead to inflammation or damage to the olfactory epithelium, impairing the transmission of olfactory signals to the brain. Consequently, individuals may experience a diminished sense of smell or even complete anosmia, contributing to a range of olfactory-related symptoms and impairments in daily life [14].

Anosmia has been identified as an initial symptom in up to 25% of COVID-19 cases, whereas xerostomia, characterized by the subjective feeling of dry mouth, has frequently been reported in COVID-19 patients and can be associated with factors directly or indirectly linked to SARS-CoV-2 [10,15]. These indirect factors encompass medications, nasal congestion, mouth breathing, nutritional deficiencies, diabetes, and the stress and anxiety induced by the pandemic or prolonged hospital stays [9,16,17,18]. Furthermore, certain studies have underscored the virus’s direct potential to invade both the peripheral and central nervous systems [19,20,21,22]. Huang et al. (2021) have substantiated this by identifying the expression of *ACE2* and *TMRPSS2* in oral mucosal epithelia and salivary glands, providing evidence of direct SARS-CoV-2 infection. Consequently, the virus’s affinity for the nervous system and the expression of host factors in both serous and mucous salivary acini, as well as taste buds, may disrupt saliva secretion, leading to xerostomia and gustatory disorders [18,21,22,23,24,25].

The primary objective of this investigation was to ascertain the prevalence of oral lesions, as well as olfactory and gustatory disorders, along with xerostomia among individuals diagnosed with COVID-19. By examining these symptoms, we aimed to contribute to the understanding of the varied manifestations of the disease, potentially shedding light on its pathophysiological mechanisms and aiding in the development of more effective diagnostic and therapeutic strategies.

## 2. Materials and Methods

This observational cohort study included a convenience sample of 414 patients, all aged 18 years or older, diagnosed with COVID-19 through reverse transcription–polymerase chain reaction assay (RT-PCR) between June 2020 and August 2021. The inclusion criteria ensured a diverse representation of individuals across different demographic groups, enhancing the generalizability of the findings. By employing rigorous diagnostic criteria, such as RT-PCR testing, we aimed to uphold the validity and reliability of our data, thus providing a solid foundation for the subsequent analysis of oral lesions and associated symptoms in the context of COVID-19 infection. The exclusion criteria were comprehensive, encompassing patients who had undergone hormone or steroid therapy within the month prior to this study, individuals using medications known to be associated with xerostomia, and those presenting with pre-existing oral lesions. These criteria were established to ensure the integrity and specificity of the study cohort, minimizing potential confounding factors that could influence the outcomes of interest.

Furthermore, adherence to ethical guidelines was paramount throughout the study process. Written informed consent, in accordance with the ethical principles outlined in the World Medical Association Declaration of Helsinki, was obtained from all participants. This ethical protocol was meticulously followed and approved by the Institutional Ethics Committee of the Brazilian Educational Association—Faculdades Unidas do Norte de Minas—Funorte, ensuring the protection of participants’ rights and the ethical conduct of the research (#46151121.6.0000.5141).

The medical history of the participants was obtained through an electronic form used in the hospital. In it, information, such as age, sex, occupation, history of smoking, and alcohol consumption of the patients, as well as the use of medications, were recorded. Information about COVID-19 infection was also collected, including the date of diagnosis, method of confirmation (PCR test, home test, antibody test, or clinical symptoms), severity of illness (mild, moderate, or severe), and presence of other pre-existing medical conditions (comorbidities). In addition, the time of onset of symptoms, such as loss of smell and taste, burning sensation, and oral dryness, was recorded in relation to the confirmation of COVID-19 infection. Possible causes of chemosensory dysfunctions, such as viral or bacterial infections, menopause, trauma to the head and neck region and surgeries in these areas, or dental surgeries, have also been recorded. More specific questions about parosmia, dysgeusia, and dysesthesia were also recorded.

The examiner was calibrated to evaluate oral lesions using the Kappa test, carried out by two independent examiners with experience in clinical dentistry and epidemiology. The Kappa test serves as a measure of agreement between two observers or two instruments that classify a series of observational units according to the classes of a qualitative variable. In this study, the percentage of agreement was >80%. Hospitalized patients in a COVID-19 referral hospital were assessed on the second and seventh days of hospitalization. The primary objective of the examination was to identify any presence of oral lesions or sensory alterations in patients and to closely monitor any progression thereof. This examination process was meticulously conducted, adhering to a systematic approach that involved the thorough examination of oral structures. Beginning with the buccal mucosa and proceeding sequentially to assess the tongue, palate, and gums, each examination step was facilitated using a sterilized wooden spatula to ensure proper hygiene and prevent cross-contamination. Direct visual inspection served as the initial method of assessment, allowing for the detection of any visible abnormalities. Additionally, a supplementary technique of gentle palpation was employed to further investigate areas of tenderness or alterations in tissue consistency, with each examination lasting approximately one minute to ensure thoroughness.

This systematic examination protocol aimed not only to detect existing oral lesions or sensory alterations but also to monitor their potential progression over time. By meticulously inspecting each oral structure in a structured order and utilizing both visual inspection and tactile palpation techniques, the examination sought to comprehensively evaluate the oral health status of patients. Through this meticulous approach, healthcare professionals could identify subtle changes in tissue appearance or texture, allowing for early detection and intervention in cases of oral pathology or sensory abnormalities.

COVID-19 symptoms were determined in accordance with the latest WHO joint report [14,26]. Questions on functional alterations were dichotomous (yes or no) and included queries, such as “do you feel the need to drink more (indicating dry mouth)?”, evaluating xerostomia and others related to olfactory and taste disorders [9]. Poisson regression was used in the research, justified by the nature of data counting, where a dependent variable represents the number of mortality events. Poisson regression is assigned to model this type of discrete variable. To analyze the associations between the variations of interest, binary logistic regression was performed. Statistical significance was identified when the *p*-value was ≤0.05.

The Wald test was employed to assess the statistical significance of coefficients in the binary logistic regression. *p*-values were reported for each coefficient, elucidating the importance of independent variables in relation to the dependent variable. Furthermore, 95% confidence intervals were provided for the estimated coefficients, demonstrating the expected range of values with a confidence level of 95%. Additionally, a significance test was conducted to evaluate the statistical significance of the constant term in the binary logistic regression model. Moreover, descriptive analyses were conducted to delineate the prevalence and characteristics of clinical manifestations among patients with COVID-19, including the determination of frequencies and proportions for various symptoms and their combinations.

The data obtained were entered into an electronic database using IBM Statistical Package for the Social Sciences^®^ (SPSS) version 24.0 software. Before statistical analysis, data were reviewed to identify and correct possible typing or coding errors. Additionally, we perform a data consistency check and exclude incomplete or inconsistent records to ensure the quality of the analyzed data.

## 3. Results

Among the 414 patients included in the study cohort, there were 208 males (50.3%) and 206 females (49.7%), reflecting a balanced gender distribution. The median age of the patients was 60.8 years, indicating a diverse age range within the sample (Supplementary Table S1). Tragically, fifty individuals (12.1%) succumbed to the illness over the course of the study period, highlighting the severity of COVID-19 outcomes observed in this population.

Utilizing Poisson regression analysis, this study revealed statistically significant associations between severe cases of COVID-19 and several key variables. Notably, comorbidities emerged as a significant factor, underscoring the impact of underlying health conditions on disease severity. Additionally, age, when dichotomized, showed a significant association with disease severity, suggesting that older individuals may be at increased risk of experiencing severe COVID-19 outcomes. Furthermore, the presence of infection-related complications was also identified as a significant predictor of disease severity, emphasizing the complex interplay between viral infection and host response mechanisms in determining clinical outcomes. These findings underscore the importance of identifying and addressing risk factors associated with severe COVID-19 illness to inform targeted interventions and improve patient outcomes.

According to Table 1, patients over 50 years of age had a 10.9% increase in mortality risk compared to those under 50 years of age (Exp(B) = 1.109, 95% CI = 1.003–1.229). Furthermore, the presence of anosmia and comorbidities increased the risk of mortality by 11.0% (Exp(B) = 1.110, 95% CI = 1.028–1.199) and 15.6% (Exp(B) = 1.156, 95% CI = 1.059–1.290), respectively. Regarding respiratory levels, patients classified as MAF (breathing monitored with an air flow sensor) presented a significantly higher risk of mortality compared to those classified as AA (breathing through room air) (Exp(B) = 1.424, 95% CI = 1.252–1.620).

These findings corroborate prior research and further substantiate the role of these factors in influencing the severity of the infection. Such evidence underscores the importance of understanding and addressing these various influences in managing and mitigating the impact of the infection. The application of binary logistic regression analysis revealed an intriguing association, and the presence of comorbidities was identified as a protective factor against death, as the presence of comorbidities was significantly associated with mortality (Wald = 5.668, *p* = 0.017, Exp(B) = 4.411, 95% CI = 1.300–14.969). Similarly, different respiration levels are also significantly associated with mortality, with increases in respiration levels correlating with a greater risk of death (respiration (1): Exp(B) = 5.027, 95% CI = 1.029–24.571; breathing (2): Exp(B) = 29.526, 95% CI = 5.697–153.018). The constant in the binary logistic regression innovation was negative and statistically significant (B = −4.370, *p* < 0.001), decreasing an inverse relationship between the constant and the occurrence of death (Table 2). This unexpected discovery prompts pertinent inquiries and underscores the intricacy of the interactions among the variables under scrutiny. It brings attention to the nuanced nature of the relationships between different factors within the context of this study, inviting further investigation and exploration to fully comprehend the underlying mechanisms at play.

More than one-third of the patients (*n* = 139, 33.5%) exhibited at least one of the COVID-19-related manifestations, comprising various symptoms. Among these, 79 individuals (19.1%) presented with oral lesions, while 75 (18.1%) reported alterations in taste perception. Additionally, 59 patients (14.2%) experienced sensations of dry mouth, commonly referred to as xerostomia, and 58 (14%) reported impairment in olfactory function (Table 3). Further analysis revealed that while the predominant manifestation was oral lesions alone in a significant proportion of cases (*n* = 40, 9.6%), there were instances where patients displayed a combination of COVID-19-associated alterations. These combinations are detailed in Table 4, indicating the complexity and variability of symptoms experienced by individuals afflicted with the virus.

The occurrence of a combination of gustatory disorder, olfactory dysfunction, xerostomia, and any form of oral lesion was identified in 18 patients, representing 4.3% of the total sample. Notably, these oral lesions manifested across a variety of anatomical sites and presented diverse clinical characteristics, as delineated in Table 5. Among these, the lips (34.2%) and tongue (27.8%) emerged as the most frequently affected areas. Within this cohort, nonspecific ulcerations constituted the predominant clinical presentation, accounting for 64.5% of cases, followed by oral candidiasis at 10.1%. This spectrum of presentations underscores the multifaceted nature of oral manifestations associated with COVID-19 and emphasizes the need for comprehensive clinical evaluation and management strategies.

## 4. Discussion

Oral mucosal lesions have emerged as prevalent complications linked to COVID-19, underscoring their importance as potential indicators of peripheral thrombosis and serving as early warning signs for potential progression to severe illness [27]. Amorim dos Santos et al. (2021) highlighted the tongue as the predominant anatomical site for oral lesions, with unspecific aphthous-like ulcers, herpes-like lesions, and fungal infections emerging as the most commonly observed clinical presentations [10]. These findings shed light on the diverse array of oral manifestations associated with COVID-19 and underscore the importance of vigilant clinical assessment and management of oral health in individuals affected by the virus. In line with the findings of Erbaş et al. (2022), which underscore the tongue as the primary location for aphthous ulcers in COVID-19 cases, our study identified the tongue (27.8%) as the second most commonly affected anatomical site, with the lips (34.2%) ranking first [28].

Etiological factors contributing to the development of aphthous lesions encompass a spectrum of influences, including stress-inducing situations, viral and bacterial infections, inflammatory states, instances of neutropenia, and occurrences of mechanical trauma. These varied factors have been observed to correlate with the onset and exacerbation of aphthous ulcers [28]. Bemquerer et al. (2020) identified ulcers (54.1%) as the most common oral lesion, alongside additional findings such as reddish macules, angina bullosa, blisters, white plaque, severe geographic and fissured tongue, petechiae, erythema multiforme-like, and necrotizing periodontal disease. It is believed that when the interaction between the virus and the ACE2 receptor occurs, it results in an alteration in the epithelial lining. This alteration increases the permeability of keratinocytes, which, in turn, facilitates the entry of pathogens. Once inside, these pathogens replicate and can lead to tissue ulceration [29].

According to the study developed by Luna-Mazzola et al. (2022), changes in tongue sensitivity were observed in patients who contracted the COVID-19 disease, evidenced by a persistent white plaque on the dorsum of the tongue and swelling in the palate and gums. In addition to the dorsum of the tongue, the labial mucosa, especially the lower lip, is often affected by COVID-19. These areas may show erosions, ulcerations, or necrotic areas, resulting in hemorrhagic crusts on the surrounding skin. Manifestations include erosive plaques, swelling, redness, and fissures, the severity of which can cause pain and affect chewing and speech [30].

Our data indicated a higher prevalence of nonspecific ulcers (64.5%), followed by candidiasis (10.1%). Specifically, among candidiasis cases, six (7.6%) were clinically identified as candidiasis pseudomembranous, while two (2.5%) exhibited candidiasis erythematous. Candidiasis was also associated with severe cases, as reported by Pergolini et al. (2023) and Kot et al.’s (2023) synthesis. The pathogenesis of candidiasis is intrinsically linked to the impairment of the immune system, either due to immunosuppressive treatments, such as antibiotic therapy, or to the disease itself, which raises the levels of pro-inflammatory mediators, disturbing tissue homeostasis and favoring the replication of opportunistic agents, such as candida. In addition, other concomitant conditions that may predispose to candidiasis have been reported, such as multiple comorbidities, inadequate oral hygiene, xerostomia, stress, local irritants, prolonged prone positioning, intubation, or denture stomatitis (in patients with poor denture hygiene). Symptoms commonly described by patients with COVID-19-associated candidiasis include pain and burning sensations, dysphagia, and the presence of characteristic membranous white patches [31,32].

According to Farid et al. (2022), nonspecific erosions and stomatitis are lesions that we provisionally consider to be true oral manifestations of COVID-19. The possible pathophysiological mechanism of erosions is associated with direct damage to the oral mucosa when SARS-CoV-2 binds to keratinocytes and non-keratinocytes. In addition, inflammation can be local or systemic, resulting in the production of inflammatory cytokines and tumor necrosis factor-alpha (TNF-α). As a consequence, neutrophil chemotaxis occurs at the site of inflammation, triggering nonspecific oral erosions and ulcerations [33].

In our comprehensive analysis, it was found that 18.1% of the patients presented with gustatory disorders, a proportion that resonates with the findings of a systematic review, underscoring the diverse prevalence rates observed across different geographical regions [34]. Interestingly, our investigation revealed a gustatory disorder prevalence falling within the spectrum observed in Latin America and Africa, further reinforcing the global variability in the manifestation of this particular symptom among individuals diagnosed with COVID-19. These findings emphasize the importance of considering regional differences when evaluating the symptomatology of COVID-19 and underscore the need for further research to elucidate the underlying factors contributing to such variations.

Xerostomia was reported by 14.2% of patients, with a similar distribution between genders. While most studies relied on questionnaires with self-reported answers to investigate xerostomia, only a small fraction objectively explored salivary flow, a more specific method for confirming hyposalivation. Although the precise mechanisms of xerostomia in COVID-19 patients are not fully understood, it has been hypothesized that alterations in saliva composition and flow could be directly caused by SARS-CoV-2 infection of the salivary glands [35]. Therefore, as observed in Saleem et al.’s (2023) study, the literature lacks objective evidence, and for a more precise analysis, it is essential to conduct objective assessments using validated, repeatable, and standardized tests to establish the frequency, extent, and cause of xerostomia in patients with COVID-19 [36].

Concerning olfactory dysfunction, we observed this alteration in 14% of patients, consistent with extensive reports in COVID-19 patients, ranging from 4.9% to 69.8% [37,38]. Interestingly, taste disorders in COVID-19 patients are often present even without accompanying smell dysfunction, with the prevalence of taste disorders frequently surpassing that of olfactory dysfunction [39]. Our results align with a study evaluating 326 Italian patients, confirming the distribution of alterations found in taste dysfunction (59.5%), xerostomia (45.9%), and olfactory dysfunctions (41.4%) [39]. Consistent with the findings of the study conducted by Fantozzi et al. (2020), gustatory dysfunction was the most commonly reported symptom (59.5%), followed by xerostomia (45.9%) and olfactory dysfunction (41.4%). Overall, 74.5% of patients with xerostomia, 78.8% of patients with gustatory dysfunction, and 71.1% of patients with olfactory dysfunction reported that all symptoms appeared prior to the diagnosis of COVID-19. Additionally, the majority of patients reported experiencing only one symptom (45.9%), while 33.3% reported a combination of two symptoms, and 20.7% reported experiencing a combination of three symptoms simultaneously [39].

According to our study, a comprehensive analysis of COVID-19 necessitates consideration of both social and biological aspects, as they each play significant roles in disease dynamics. In the social sphere, studies by Ahmed et al. (2020) and Simões and Silva (2020) have highlighted factors, such as age, gender, ethnicity, socioeconomic status, and access to health services, all of which profoundly influence virus exposure, disease severity, and health outcomes [40,41]. Marginalized groups, in particular, may encounter additional barriers to accessing adequate healthcare, thereby heightening their risk of infection and experiencing severe complications. Moreover, behaviors like adherence to preventive measures and social distancing directly impact disease transmission.

From a biological standpoint, our study identified individual characteristics such as age, pre-existing comorbidities, and immune health factors as critical determinants of infection susceptibility and symptom severity. These findings align with research conducted by Docherty et al. (2020), which underscores that older individuals or those with underlying medical conditions, such as diabetes or cardiovascular disease, face an elevated risk of developing severe complications from COVID-19 [42]. Additionally, variations in immune response among individuals, influenced by genetic and immunological factors, significantly contribute to disease progression and recovery. Therefore, a comprehensive understanding of COVID-19’s impact on both public and individual health necessitates consideration of both social and biological dimensions to inform effective prevention, diagnosis, and treatment strategies.

While acknowledging the convenience of our sample, it is imperative to recognize the inherent limitations within our study. Firstly, the data collection method utilized dichotomous questions, allowing for a simple yes or no response, which may have inadvertently overlooked nuances in experiences of xerostomia, olfactory, and gustatory dysfunctions. This limitation could potentially undermine the comprehensiveness of our findings, as the complexity of these symptoms may not have been fully captured. Additionally, the timing of our survey, conducted shortly after COVID-19 diagnoses, introduces a temporal factor that could influence the accuracy of reported symptoms. The acute phase of the illness may have led to variations in symptom severity and perception, impacting the reliability of our data.

Moreover, the influence of medications used in the treatment of COVID-19 on oral health and xerostomia cannot be disregarded. Certain medications may have side effects that manifest as oral alterations or exacerbate pre-existing conditions, such as dry mouth. This aspect adds another layer of complexity to our findings, as it suggests a potential confounding variable that may not have been fully accounted for in our analysis. Furthermore, the scarcity of literature surrounding COVID-19, given its relatively recent emergence, contributes to the overarching limitations of our study. The lack of extensive prior research restricts our ability to contextualize and compare our findings, highlighting the need for further investigation into the oral manifestations of COVID-19 and their implications for clinical practice.

## 5. Conclusions

Our study highlights the diverse clinical manifestations and prognostic factors associated with COVID-19. Significant associations were found between severe COVID-19 cases and variables such as comorbidities, age, and infection-related complications. Unexpectedly, the presence of comorbidities was identified as a protective factor against death in logistic regression analysis, adding complexity to the understanding of disease outcomes. Additionally, respiratory levels were significantly associated with mortality, underlining the importance of respiratory status in predicting outcomes. A significant proportion of patients exhibited COVID-19-related manifestations, with oral lesions being the most common. These findings underscore the complexity of COVID-19 clinical presentations and the need for comprehensive management strategies to improve patient outcomes. Further research is warranted to elucidate underlying mechanisms and develop targeted interventions for effective management of COVID-19 complications.

## Figures and Tables

**Table 1:** Parameter estimates and associations with mortality in a poisson regression analysis.

Parameter Estimates										
Parameter	B	Erro Padrão	95% Wald Confidence Interval		Hypothesis Testing			Exp(B)	95% Wald Confidence Interval for Exp(B)	
			Lower	Upper	Wald Chi-Square	df	Sig.		Lower	Upper
(Intercept)	−0.250	0.0765	−0.400	−0.100	10.688	1	0.001	0.779	0.670	0.905
[Age_dico = 2]	0.104	0.0520	0.003	0.206	4.038	1	0.044	1.110	1.003	1.229
[Age_dico = 1]	0 ^a^	-	-	-	-	-	-	1	-	-
[Anosmia = 2]	0.105	0.0394	0.027	0.182	7.052	1	0.008	1.110	1.028	1.199
[Anosmia = 1]	0 ^a^	-	-	-	-	-	-	1	-	-
[Comorbidity = 2]	0.156	0.0503	0.057	0.255	9.604	1	0.002	1.169	1.059	1.290
[Comorbidity = 1]	0 ^a^	-	-	-	-	-	-	1	-	-
[Breathing = 3]	0.354	0.0658	0.225	0.483	28.894	1	0.000	1.424	1.252	1.620
[Breathing = 2]	0.128	0.0496	0.031	0.225	6.671	1	0.010	1.137	1.031	1.253
[Breathing = 1]	0 ^a^	-	-	-	-	-	-	1	-	-
(Scale)	1 ^b^									

Dependent variable: death; model: (intercept), age_dico, anosmia, comorbidity, breathing. ^a^. Set to zero because this parameter is redundant. ^b^. Fixed at the displayed value. Encoding: age: 1 (up to 50 years old) and 2 (over 50 years old); anosmia: 1 (yes) and 2 (no); comorbidities: 1 (yes) and 2 (no); breathing: 1 (AA), 2 (CN), and 3 (MAF). AA: ambient air, CN: nasal cannula, and MAF: airflow sensor.

**Table 2:** Binary logistic regression between the risk or absence of death from COVID-19 and independent variables.

Variables in the Equation									
		B	S.E.	Wald	df	Sig.	Exp(B)	95% C.I. for EXP(B)	
								Lower	Upper
Step 1	Comorbidity (1)	1.484	0.623	5.668	1	0.017	4.411	1.300	14.969
	Breathing			19.901	2	0.000			
	Breathing (1)	1.615	0.810	3.979	1	0.046	5.027	1.029	24.571
	Breathing (2)	3.385	0.839	16.263	1	0.000	29.526	5.697	153.018
	Constant	−4.370	0.926	22.292	1	0.000	0.013		

Variable(s) entered in step 1: comorbidities, breathing.

**Table 3:** Oral lesions, olfactory and gustatory disorders, and xerostomia in patients with COVID-19.

Clinical Manifestations	*n*	%	Male	%	Female	%
Oral lesions	79	19.08	36	45.56	43	54.43
Gustatory disorders	75	18.11	40	53.33	35	46.66
Xerostomia	59	14.25	30	50.84	29	49.15
Olfactory dysfunction	58	14.00	27	46.55	31	53.44
TOTAL	139/414					

**Table 4:** The distribution of oral lesions and sensorial functions alterations by the number of patients.

Clinical Manifestations	*n*	%
Oral lesions only	40	9.66
Gustatory disorders, olfactory dysfunction, xerostomia, and oral lesions	18	4.34
Gustatory disorders and olfactory dysfunction	16	3.86
Gustatory disorders, olfactory dysfunction, and xerostomia	15	3.62
Gustatory disorders	14	3.38
Xerostomia and oral lesions	11	2.65
Xerostomia	11	2.65
Gustatory disorders, olfactory dysfunction, and oral lesions	7	1.69
Gustatory disorders and oral lesions	3	0.72
Gustatory disorders and xerostomia	2	0.48
Olfactory dysfunction and xerostomia	2	0.48
TOTAL	139/414	33.57

**Table 5:** Location and clinical presentations of types of oral lesions.

Anatomic Location	*n*	%	Types of Oral Lesions	*n*	%
Unspecific lips	27	34.17	Ulcerations	51	64.55
Tongue	22	27.84	Candidiasis	8	10.12
Other nonspecific mucosal sites	16	20.25	Erythema or red plaques	7	8.86
Labial commissure	8	10.12	Herpes-like lesions	5	6.32
Gingiva	4	5.06	Blisters	5	6.32
Palate	2	2.53	Lingual papilites	3	3.79
TOTAL	79	100		79	100

## Data Availability

The raw data supporting the conclusions of this article will be made available by the authors on request.

## Supplementary Materials

The following supporting information can be downloaded at https://www.mdpi.com/article/10.3390/dj12060179/s1, Table S1: The clinical characteristics of the patients who presented with symptoms, such as age, sex, history of smoking or alcohol, any medications.

## Abbreviations

COVID-19: coronavirus disease; SARS-CoV-2: respiratory syndrome coronavirus-2; ACE2: angiotensin-converting enzyme 2; RT-PCR: transcription–polymerase chain reaction assay; SPSS: *Statistical Package for the Social Sciences^®^*.
