# Supplementary material for: Frequency of Oral Lesions, Olfactory, and Gustatory Disorders and Xerostomia in Patients with COVID-19
Source: Dent J (Basel). 2024 Jun 11;12(6):179. doi: 10.3390/dj12060179 (PMC11203208; doi:10.3390/dj12060179)
Supplement: Supplementary file 1 [file dentistry-12-00179-s001.zip › dentistry-2771836-supplementary.pdf]

Supplementary Table S1. The clinical characteristics of the patients who presented with symptoms, such as age, sex, history of smoking or alcohol, any medications.

Patient	Sex	Age	History of smoking or alcohol	Medicines	Comorbidities
1.	M	54	NR	NR	NR
2.	M	29	NR	NR	NR
3.	F	90	NR	NR	NR
4.	M	59	NR	NR	NR
5.	F	57	NR	Eszopiclone 3mg, Losartan 50mg, hydroxychloroquine 400mg, Tenoxicam 20mg, Diacerein 60mg, Triamcinolone 3mg and Famotidine 60mg	Rheumatoid arthritis, Sjogren's syndrome and AH
6.	M	58	NR	Prednisolone, Acetylfuroxime, Formoterol, Heparin, Dexamethasone and Omeprazole	NR
7.	M	66	NR	NR	NR
8.	M	28	NR	Insulin NPH 30UI, Insulin regular 15UI, Olmesartan Medoxomil + Amlodipine 40, Ibuprofen, Amoxicillin + Clavulanate 500 + 125, Azithromycin, Ivermectin, Vitamin C, Vitamin D and Zinc	AH and DM2
9.	M	83	NR	Selozok and Aradois	AH
10.	M	59	NR	Ceftriaxone, Azithromycin, Dexamethasone, Colchicine and Piperacillin Sodium + Tazobactam Sodium	NR
11.	M	77	NR	NR	NR
12.	M	32	NR	Ceftriaxone, Azithromycin, Piperacillin Sodium + Tazobactam Sodium, Dexamethasone and Colchicine	AH and DM2
13.	F	82	NR	NR	AH, DM2 and hypothyroidism
14.	F	70	NR	Losartan, Atenolol and Clonazepam	AH
15.	M	75	NR	Somalgin 80mg, Simvastatin 40mg, Losartan 50mg, Metoprolol 50mg, Azithromycin, Dexamethasone, Piperacillin Sodium + Tazobactam Sodium and	AH

				Colchicine	
16.	F	74	NR	Xarelto, Digoxin, Hydrochlorothiazide , Captopril, Bisoprolol, Spiroinolactone, Hydralazine, Monocordyl and Anlodipine	AH, DM2, congestive heart failure and chronic arterial fibrillation
17.	F	69	NR	NR	AH and DM2
18.	F	80	NR	Olmeclore 40mg, Pantoprazole 40mg, Metoprolol succinate 50mg, Brinonidine eye drops and Ceftriaxone	AH
19.	M	86	NR	NR	Alzheimer
20.	M	84	NR	NR	NR
21.	M	76	Long smoker	Losartan 25mg, Clopidogrel 75mg, Bisoprolol 5mg, Simvastatin 20mg, Spironolactone 25mg, Furosemide 20mg, Aspirin 100mg and Salbutamol 100mcg	AH
22.	F	61	NR	NR	NR
23.	M	59	NR	NR	NR
24.	F	73	NR	Losartan, Hydrochlorothiazide, Azithromycin and Ceftriaxone	AH
25.	F	73	NR	Losartan and Hydrochlorothiazide	AH
26.	M	69	NR	NR	NR
27.	F	77	NR	Pyrazinamide	TUBERCULOSIS
28.	F	65	NR	Glifage XR 500mg, Losartan 50mg and Hydrochlorothiazide 25mg	AH and DM2
29.	F	65	NR	NR	NR
30.	M	45	NR	NR	NR
31.	F	93	NR	Ceftriaxone, Cefepime, Azithromycin, Dexamethasone and Enoxaparin	AH, nephropathic renal insufficiency
32.	M	53	NR	NR	AH and epilepsy
33.	F	47	NR	NR	AH and hypothyroidism
34.	F	65	NR	Omeprazole 20mg, Carvedilol 3,125mg, Metformin 850mg,	AH and hypothyroidism

				Ciprofibrate 100mg, Simvastatin 20mg, and Calcium carbonate sodium alendronate 70mg	
35.	F	77	NR	NR	NR
36.	F	64	NR	NR	NR
37.	F	64	NR	NR	NR
38.	M	66	NR	NR	AH and DM2
39.	M	63	NR	NR	NR
40.	F	83	NR	NR	NR
41.	F	64	NR	Azithromycin, Ceftriaxone, Oseltamivir, Azithromycin, Ceftriaxone and Oseltamivir	NR
42.	F	71	NR	NR	NR
43.	M	55	NR	NR	NR
44.	F	67	NR	NR	NR
45.	F	67	NR	NR	NR
46.	F	66	NR	NR	NR
47.	M	81	NR	NR	NR
48.	M	81	NR	NR	NR
49.	M	47	NR	NR	NR
50.	F	64	NR	NR	NR
51.	M	---	NR	NR	NR
52.	M	63	NR	NR	NR
53.	M	75	NR	Losartan, Carvedilol, Amlodipine, Simvastatin, Glifage and Gliclazide	AH and DM2
54.	F	68	NR	NR	NR
55.	F	67	NR	AZITHROMYCIN CEFTRIAXONE OSELTAMIVIR DEXAMETHASONE - PIPER TAZO	DM2, hypertension and dyslipidemia
56.	64	67	NR	NR	NR
57.	M	68	Smoker	NR	NR
58.	F	78	NR	NR	NR
59.	F	76	NR	Mirtazapine 15mg, Bisoprolol 25mg, Levothyroxine 50mcg, Alenia (400/12mcg) and Spiriva (25mcg)	AH
60.	F	67	NR	NR	NR
61.	F	82	NR	NR	AH, hypothyroidism, and heart failure
62.	M	89	NR	NR	NR
63.	M	73	NR	Losartan 50mg, Selozok 50mg, Furosemide 40mg and hydrochlorothiazide 25mg	AH
64.	F	80	NR	Losartan 50mg, Hydrochlorothiazide 25 mg, and Simvastatin 20mg	AH
65.	M	68	NR	NR	NR

66.	M	69	NR	NR	NR
67.	M	62	NR	NR	NR
68.	F	51	NR	NR	NR
69.	F	62	NR	NR	NR
70.	M	70	NR	NR	NR
71.	F	96	NR	NR	NR
72.	M	63	NR	NR	NR
73.	M	27	NR	NR	NR
74.	M	70	NR	NR	NR
75.	M	81	NR	NR	NR
76.	M	74	NR	NR	NR
77.	F	64	NR	NR	NR
78.	F	60	NR	NR	NR
79.	M	86	NR	NR	NR
80.	M	74	NR	NR	NR
81.	M	56	NR	NR	NR
82.	M	56	NR	NR	NR
83.	F	56	NR	NR	NR
84.	F	92	NR	NR	NR
85.	M	72	NR	NR	NR
86.	F	35	NR	NR	NR
87.	M	48	NR	NR	NR
88.	M	53	NR	NR	NR
89.	F	41	NR	NR	NR
90.	F	73	NR	NR	NR
91.	M	60	NR	NR	NR
92.	M	81	NR	NR	NR
93.	F	80	NR	NR	NR
94.	F	60	NR	NR	NR
95.	F	51	NR	NR	NR
96.	M	41	NR	NR	NR
97.	F	54	NR	NR	NR
98.	M	67	NR	NR	NR
99.	M	66	NR	NR	NR
100.	M	46	NR	NR	NR
101.	F	46	NR	NR	NR
102.	M	35	NR	NR	NR
103.	M	42	NR	NR	NR
104.	M	84	NR	NR	NR
105.	F	64	NR	NR	NR
106.	M	77	NR	NR	NR
107.	F	77	NR	NR	NR
108.	F	77	NR	NR	NR
109.	F	41	NR	NR	NR
110.	F	39	NR	NR	NR
111.	F	46	NR	NR	NR
112.	M	62	NR	NR	NR
113.	M	97	NR	NR	NR
114.	F	69	NR	NR	NR

115.	F	38	NR	NR	NR
116.	M	37	NR	NR	NR
117.	F	44	NR	NR	NR
118.	M	72	NR	NR	NR
119.	F	80	NR	NR	NR
120.	M	97	NR	NR	NR
121.	F	84	NR	NR	NR
122.	M	95	NR	NR	NR
123.	F	87	NR	NR	NR
124.	M	79	NR	NR	NR
125.	F	74	NR	NR	NR
126.	M	63	NR	NR	NR
127.	F	84	NR	NR	NR
128.	M	53	NR	NR	NR
129.	M	78	NR	NR	NR
130.	M	60	NR	NR	NR
131.	M	46	NR	NR	NR
132.	M	49	NR	NR	NR
133.	F	38	NR	NR	NR
134.	M	38	NR	NR	NR
135.	F	75	NR	NR	NR
136.	F	54	NR	NR	NR
137.	F	94	NR	NR	NR
138.	M	58	NR	NR	NR
139.	F	44	NR	NR	NR
140.	M	87	NR	NR	NR
141.	M	89	NR	NR	NR
142.	F	89	NR	NR	NR
143.	F	37	NR	NR	NR
144.	F	44	NR	NR	NR
145.	F	81	NR	NR	NR
146.	M	18	NR	NR	NR
147.	M	53	NR	NR	NR
148.	F	30	NR	NR	NR
149.	M	85	NR	NR	NR
150.	F	81	NR	NR	NR
151.	M	42	NR	NR	NR
152.	M	34	NR	NR	NR
153.	M	79	NR	NR	NR
154.	F	59	NR	NR	NR
155.	M	80	NR	NR	NR
156.	M	82	NR	NR	NR
157.	M	50	NR	NR	NR
158.	M	67	NR	NR	NR
159.	F	54	NR	NR	NR
160.	F	54	NR	NR	NR
161.	F	23	NR	NR	NR
162.	M	67	NR	NR	NR
163.	M	73	NR	NR	NR

164.	M	42	NR	NR	NR
165.	F	60	NR	NR	NR
166.	M	71	NR	NR	NR
167.	M	61	NR	NR	NR
168.	F	66	NR	NR	NR
169.	M	70	NR	NR	NR
170.	F	61	NR	NR	NR
171.	F	93	NR	NR	NR
172.	M	39	NR	NR	NR
173.	M	48	NR	NR	NR
174.	F	90	NR	NR	NR
175.	F	81	NR	NR	NR
176.	M	42	NR	NR	NR
177.	M	78	NR	NR	NR
178.	M	79	NR	NR	NR
179.	M	52	NR	NR	NR
180.	M	35	NR	NR	NR
181.	M	47	NR	NR	NR
182.	M	47	NR	NR	NR
183.	M	86	NR	NR	NR
184.	M	36	NR	NR	NR
185.	F	77	NR	NR	NR
186.	M	75	NR	NR	NR
187.	M	66	NR	Losartan, Clopidogrel, Insulin NPH, Simvastatin and Leflunomide	AH and DM2
188.	M	65	NR	Metformin, Losartan and Simvastatin	AH
189.	M	54	NR	NR	NR
190.	M	47	NR	NR	AH and DM2
191.	M	92	NR	NR	NR
192.	M	67	NR	NR	NR
193.	F	62	NR	NR	NR
194.	F	69	NR	Losartan, Insulin and Metformin	AH and DM2
195.	F	68	NR	Clonazepam	AH
196.	F	75	NR	NR	AH and DM2
197.	F	76	NR	NR	AH and DM2
198.	F	35	NR	NR	NR
199.	F	42	NR	NR	NR
200.	F	76	NR	NR	Schizophrenia
201.	F	80	NR	NR	NR
202.	F	58	NR	NR	NR
203.	F	61	NR	NR	NR
204.	F	58	NR	NR	NR
205.	F	61	NR	NR	NR
206.	F	85	NR	NR	NR
207.	F	53	NR	NR	AH and DM2
208.	F	67	NR	NR	AH and DM2
209.	F	56	NR	NR	DOWN'S

					SYNDROME
210.	F	65	NR	NR	AH and DM2
211.	M	53	NR	NR	NR
212.	F	41	NR	NR	NR
213.	F	93	NR	NR	NR
214.	M	45	NR	NR	AH and DM2
215.	M	68	NR	NR	AH and DM2
216.	F	70	NR	NR	AH and DM2
217.	F	78	NR	NR	AH and DM2
218.	M	60	NR	NR	NR
219.	M	62	NR	NR	DM2
220.	M	93	NR	NR	NR
221.	M	53	NR	NR	NR
222.	M	60	NR	NR	NR
223.	F	52	NR	NR	NR
224.	F	45	NR	NR	NR
225.	M	71	NR	NR	AH and DM2
226.	F	57	NR	NR	AH and DM2
227.	F	66	NR	NR	HYPOTHYROIDISM
228.	F	63	NR	NR	AH and DM2
229.	M	37	NR	NR	AH andDM2
230.	M	87	NR	NR	NR
231.	F	42	NR	NR	NR
232.	F	85	NR	NR	NR
233.	F	31	NR	NR	NR
234.	F	49	NR	NR	NR
235.	F	44	NR	NR	NR
236.	F	32	NR	NR	NR
237.	F	39	NR	Atenolol and Hydrochlorothiazide	AH
238.	F	62	NR	NR	NR
239.	F	68	NR	NR	NR
240.	F	82	NR	NR	NR
241.	M	52	NR	NR	NR
242.	M	88	NR	NR	NR
243.	F	62	NR	NR	NR
244.	M	62	NR	NR	NR
245.	M	42	NR	NR	NR
246.	M	55	NR	NR	NR
247.	F	48	NR	NR	NR
248.	M	20	NR	NR	NR
249.	M	63	NR	Cefepime, Clarithromycin, Dexamethasone, Colchicine and Methylprednisolone	NR
250.	F	79	NR	Ceftriaxone<br>Clarithromycin d2<br>Cefepime d	NR
251.	F	47	NR	Hydrochlorothiazide	AH

252.	F	32	NR	NR	NR
253.	F	84	NR	Xarelto 10mg, Carvedilol 125mg, Clopidogrel 75mg, Furosemide 40mg, Symbicort spray glycopirronum 50 mcg, Mirtazapine 30 mg	NR
254.	M	47	NR	NR	NR
255.	F	57	NR	Losartan 50mg	AH
256.	F	69	NR	Losartan 50mg, Amlodipine 5mg, Selozok, Aspirin 100mg mid, Clopidogrel 75, Rosuvastatin 20mg, Indapamide 15mg, Monocordil 20mg, Metformin and Ivabradine 5mg	AH and DM2
257.	M	66	NR	Clarithromycin, Cefepime, Ceftriaxone, Methylprednisolone - prednisone 20mg, Colchicine d5 and Ibuprofen D5	NR
258.	M	82	NR	Carvedilol 3125mg, Furosemide 40mg, Spironolactone 25mg, Captopril 125mg and Edoxaban 30mg	AH and DM2
259.	M	29	NR	NR	NR
260.	M	26	NR	NR	NR
261.	F	59	NR	Losartan 50mg	AH
262.	M	60	NR	NR	NR
263.	M	48	NR	NR	NR
264.	M	82	NR	Furosemide	Chronic obstructive pulmonary disease
265.	F	55	NR	NR	NR
266.	F	37	NR	NR	NR
267.	F	49	NR	NR	NR
268.	M	70	NR	NR	NR
269.	F	40	NR	NR	NR
270.	F	64	NR	NR	AH
271.	F	66	NR	NR	AH and DM2
272.	F	36	NR	NR	NR
273.	F	47	NR	NR	NR
274.	F	62	NR	NR	NR
275.	F	41	NR	NR	NR
276.	F	32	NR	NR	NR
277.	M	51	NR	NR	AH and DM2
278.	M	56	NR	NR	AH and DM2
279.	M	41	NR	NR	AH and DM2
280.	M	27	NR	NR	NR
281.	M	62	NR	NR	AH and DM2
282.	M	56	NR	NR	AH and DM2
283.	M	72	NR	NR	NR

284.	M	69	NR	NR	NR
285.	F	47	NR	NR	NR
286.	F	63	NR	NR	AH and DM2
287.	F	73	NR	NR	AH and DM2
288.	F	92	NR	NR	NR
289.	F	37	NR	NR	NR
290.	F	70	NR	NR	AH and DM2
291.	F	41	NR	NR	NR
292.	M	64	NR	NR	NR
293.	F	71	NR	NR	AH
294.	F	48	NR	NR	AH
295.	M	66	NR	NR	AH
296.	M	47	NR	NR	AH
297.	M	26	NR	NR	NR
298.	M	52	NR	NR	NR
299.	M	61	NR	NR	NR
300.	M	42	NR	NR	NR
301.	M	36	NR	NR	NR
302.	M	63	NR	NR	AH
303.	M	33	NR	NR	NR
304.	M	57	NR	NR	AH
305.	M	51	NR	NR	AH
306.	M	89	NR	NR	NR
307.	M	36	NR	NR	NR
308.	M	41	NR	NR	NR
309.	F	31	NR	NR	NR
310.	F	43	NR	NR	AH
311.	F	78	NR	NR	NR
312.	F	85	NR	NR	NR
313.	F	65	NR	NR	AH
314.	F	76	NR	NR	NR
315.	F	52	NR	NR	AH
316.	M	42	NR	NR	AH and DM2
317.	M	90	NR	NR	NR
318.	F	67	NR	NR	NR
319.	F	63	NR	NR	AH
320.	M	58	NR	NR	AH
321.	M	37	NR	NR	NR
322.	M	30	NR	NR	NR
323.	F	40	NR	Losartan	AH
324.	F	42	NR	Losartan	AH
325.	F	51	NR	Losartan	AH
326.	M	75	NR	NR	NR
327.	F	81	NR	NR	NR
328.	F	30	NR	NR	NR

329.	F	72	NR	NR	NR
330.	M	51	NR	NR	NR
331.	F	41	NR	NR	NR
332.	F	52	NR	NR	NR
333.	F	47	NR	NR	NR
334.	F	51	NR	NR	NR
335.	M	41	NR	NR	NR
336.	F	78	NR	NR	NR
337.	M	55	NR	Losartan	AH
338.	M	36	NR	Losartan	AH
339.	F	67	NR	NR	NR
340.	M	43	NR	NR	NR
341.	M	45	NR	Losartan	AH
342.	M	43	NR	NR	NR
343.	F	67	NR	NR	NR
344.	M	35	NR	NR	NR
345.	F	51	NR	NR	NR
346.	F	68	NR	NR	NR
347.	F	84	NR	NR	AH and DM2
348.	F	36	NR	NR	AH and DM2
349.	M	43	NR	NR	AH
350.	M	59	NR	NR	AH
351.	M	40	NR	NR	NR
352.	M	57	NR	NR	AH
353.	M	75	NR	NR	AH
354.	F	41	NR	NR	NR
355.	F	32	NR	NR	NR
356.	F	63	NR	Clonazepam	Hypothyroidism
357.	F	41	NR	NR	NR
358.	M	54	NR	NR	NR
359.	M	27	NR	NR	NR
360.	M	58	NR	NR	NR
361.	M	57	NR	NR	AH
362.	M	57	NR	NR	AH
363.	M	46	NR	NR	AH
364.	F	45	NR	NR	AH
365.	F	35	NR	NR	AH
366.	F	89	NR	NR	AH
367.	M	38	NR	NR	DM2
368.	M	38	NR	NR	NR
369.	M	54	NR	NR	AH
370.	F	89	NR	NR	NR
371.	M	45	NR	NR	NR

372.	F	87	NR	NR	NR
373.	F	64	NR	NR	AH, DM2 fibromyalgia and Chronic obstructive pulmonary disease
374.	F	63	NR	Losartan	AH
375.	F	91	NR	NR	NR
376.	F	87	NR	Clopidogrel 75mg, Diovan anlo fix, Lasix 40mg, Pantoprazole 40mg, Atorvastatin 20mg, Nebivolol 5mg, Procolan 75mg, Domperidone 10mg, Alenia 12/400mg, Prosso km, Osteotec	AH and cardiopathy
377.	F	37	NR	NR	NR
378.	M	68	NR	NR	AH and DM2
379.	M	38	NR	Losartan 50mg, Hydrochlorothiazide 24mg, Theophylline and Quetiapine	AH and lymphadenopathy
380.	M	33	NR	NR	NR
381.	F	35	NR	Ritalin, Clonazepam and Pregabalin	AH
382.	F	91	NR	NR	NR
383.	M	40	NR	NR	NR
384.	M	35	NR	Losartan 50mg	AH
385.	M	35	NR	NR	NR
386.	M	65	NR	NR	NR
387.	M	68	NR	NR	NR
388.	M	73	NR	NR	NR
389.	F	41	NR	NR	NR
390.	F	82	NR	Losartan, Furosemide, Carvedilol, Gliclazide, Metformin, Simvastatin, NPH insulin	AH and adenoidplasty
391.	F	80	NR	Indapamide e Losartan	AH
392.	M	35	NR	NR	NR
393.	F	66	NR	Losartan risperidone	AH and Chagas diseases
394.	M	30	NR	NR	NR
395.	F	39	NR	NR	NR
396.	M	46	NR	Losartan 50mg	AH
397.	M	53	NR	Losartan 50mg	AH
398.	F	44	NR	Fentanyl 35ml, Midazolam 35ml, Atracurium 20 and Propofol 30	AH

399.	F	61	NR	NR	NR
400.	F	81	NR	NR	NR
401.	F	52	NR	NR	NR
402.	M	32	NR	NR	AH
403.	F	80	NR	NR	NR
404.	F	48	NR	NR	AH and obesity
405.	M	27	NR	NR	NR
406.	M	68	NR	Losartan 50mg	AH
407.	M	77	NR	NR	NR
408.	M	87	NR	Losartan 50mg	AH and Alzheimer's
409.	M	54	NR	NR	NR
410.	F	49	Smoker for 49 years	NR	AH, obesity and chronic obstructive pulmonary disease
411.	F	85	NR	NR	NR
412.	F	63	Long time smoker, smokes approximately 14 cigarettes a day	Olanzapine 10mg, Escitalopram 10mg, Pregabalin 75mg	AH and chronic obstructive pulmonary disease
413.	F	75	Alcoholic	NR	AH, DM2 and Alzheimer's
414.	F	62	NR	Losartan, Hydrochlorothiazide and Metformin	AH and DM2

NR: Not reported; AH: Arterial hypertension, DM: Diabetes mellitus, DM2: Diabetes mellitus type 2
